# Estimating the Population Impact of a New Pediatric Influenza Vaccination Program in England Using Social Media Content

**DOI:** 10.2196/jmir.8184

**Published:** 2017-12-21

**Authors:** Moritz Wagner, Vasileios Lampos, Elad Yom-Tov, Richard Pebody, Ingemar J Cox

**Affiliations:** ^1^ Public Health England London United Kingdom; ^2^ University College London London United Kingdom; ^3^ London School of Hygiene and Tropical Medicine London United Kingdom; ^4^ Department of Computer Science University College London London United Kingdom; ^5^ Microsoft Research Herzliya Israel; ^6^ Department of Computer Science University of Copenhagen Copenhagen Denmark

**Keywords:** health intervention, influenza, vaccination, social media, Twitter

## Abstract

**Background:**

The rollout of a new childhood live attenuated influenza vaccine program was launched in England in 2013, which consisted of a national campaign for all 2 and 3 year olds and several pilot locations offering the vaccine to primary school-age children (4-11 years of age) during the influenza season. The 2014/2015 influenza season saw the national program extended to include additional pilot regions, some of which offered the vaccine to secondary school children (11-13 years of age) as well.

**Objective:**

We utilized social media content to obtain a complementary assessment of the population impact of the programs that were launched in England during the 2013/2014 and 2014/2015 flu seasons. The overall community-wide impact on transmission in pilot areas was estimated for the different age groups that were targeted for vaccination.

**Methods:**

A previously developed statistical framework was applied, which consisted of a nonlinear regression model that was trained to infer influenza-like illness (ILI) rates from Twitter posts originating in pilot (school-age vaccinated) and control (unvaccinated) areas. The control areas were then used to estimate ILI rates in pilot areas, had the intervention not taken place. These predictions were compared with their corresponding Twitter-based ILI estimates.

**Results:**

Results suggest a reduction in ILI rates of 14% (1-25%) and 17% (2-30%) across all ages in only the primary school-age vaccine pilot areas during the 2013/2014 and 2014/2015 influenza seasons, respectively. No significant impact was observed in areas where two age cohorts of secondary school children were vaccinated.

**Conclusions:**

These findings corroborate independent assessments from traditional surveillance data, thereby supporting the ongoing rollout of the program to primary school-age children and providing evidence of the value of social media content as an additional syndromic surveillance tool.

## Introduction

### Background

In 2012 the Joint Committee on Vaccination and Immunisation recommended the extension of the annual influenza vaccination campaign to include all healthy children aged 2 to 16 years of age in England [1]. This decision was informed by influenza transmission modeling done using an evidence-synthesis approach, showing that vaccination could not only protect the children themselves from infection, but also decrease influenza transmission in the general population. This finding included the indirect protection of at-risk groups, such as people over 65 years of age or those with underlying clinical risk factors [2]. The phased rollout of the live attenuated influenza vaccine (LAIV) program began during the 2013/2014 influenza season. In the first season, the program offered vaccinations to all 2 and 3-year-olds throughout England. A number of geographically distinct pilot regions also offered vaccinations to primary school age children (4-11 years of age) to determine the optimal model of delivery to school-age children. For the 2014/2015 influenza season, the program was extended nationally to offer vaccinations to all 2 to 4-year-olds. Pilot locations were added that offered vaccinations to children either (1) of primary school age (*Primary school*; 4-11 years), (2) the first two years of secondary school age (*Secondary school*, 11-13 years), or (3) both (*Primary and Secondary school*; 4-13 years) to determine optimal models of delivery.

### Motivation

Public Health England (PHE) has been using a variety of surveillance systems to assess the overall population impact of the childhood influenza campaign in children of school-age on influenza epidemiology to validate the direct and indirect effects of vaccinating this age group. The pilot locations for 2014/2015 are of particular interest, as the variation in target groups may offer further insights into the optimal strategies for the national rollout. During the 2014/2015 campaign, most influenza indicators through traditional surveillance systems in both targeted and nontargeted age groups demonstrated a significant reduction in pilot areas that offered the vaccine to primary school age children. However, there was little impact in pilot areas, where only two age cohorts of secondary school age children were vaccinated [3]. These surveillance indicators were based on health systems ranging from General Practitioners’ consultation rates to excess mortality.

Whilst such results are important in estimating the intervention’s effects on health care services, online user-generated information offers a complementary data source that can provide additional insights into the impact of such campaigns on the wider community, including those persons that do not consult the health care system. Our study also highlights the potential value of user-generated information in the absence of routine evaluation systems. Internet-based surveillance systems are being viewed as novel logistically and economically viable developments that offer great potential as an extension of traditional surveillance systems [4]. Recent research efforts have shown that in combination with *machine learning* techniques, data from social media or search engines can be used to accurately estimate disease-related indicators such as influenza-like illness (ILI) rates [5-9]. These technologies provide health monitoring systems with additional, publicly available, and potentially more timely sources of data for syndromic surveillance. Furthermore, compared to traditional surveillance systems, user-generated content may offer insights about a wider range of the population, including the bottom part of the disease population pyramid (ie, those that do not seek medical attention) [10].

For the 2013/2014 pilot areas, in order to provide further evidence of the community-wide effects of vaccinating children with influenza vaccine, Lampos et al made use of online user-generated content in combination with statistical natural language processing techniques to estimate ILI rates in the population [9]. By matching nonvaccinated control areas with pilot areas and using flu-related Twitter posts or Bing search queries from these locations, the impact of the campaign within the *Primary school* age pilot areas was estimated, showing a significant decrease (22% to 33% reduction) in influenza transmission in the general population in these pilot areas compared to corresponding control areas [9]. PHE’s estimates also showed evidence of a reduction in influenza transmission in targeted and nontargeted age groups in pilot areas compared to nonpilot areas, based on a variety of influenza indicators during a season dominated by circulation of influenza A(H1N1)pdm09 [11].

### Aim

The work in this paper applies the same statistical framework as Lampos et al [9] (with a slightly improved supervised learning approach) on Twitter data for the influenza season of 2014/2015. We aim to assess the impact of influenza vaccine pilot trials in school age children on influenza transmission in those pilot areas. The 2014/2015 season was dominated by circulation of influenza A(h3N2) and influenza B. In addition, we examined the impact of vaccinating different target populations, specifically primary and/or secondary school-age children, on influenza rates in the general population. This analysis provides further insights into the most effective strategies for reducing community-wide influenza transmission. This work also aims to reevaluate the hypothesis that a statistical framework based on online user-generated content can form a valid source for more fine-grained influenza surveillance tasks, such as estimating the impact of a targeted intervention. We repeated the analysis for the 2013/2014 LAIV campaign that was previously studied in Lampos et al [9], but with revised pilot and control areas, for consistency with our study for the 2014/2015 season.

## Methods

### Data Sources

Two data sources were used for the experiments: geo-located Twitter posts related to ILI and official ILI rates provided by the Royal College of General Practitioners (RCGP) [12], the latter defining the *ground truth*. In addition, boundary data and population estimates from the Office for National Statistics (ONS) [13,14] were used to map the vaccine pilot and control areas.

#### Twitter Data

The Twitter data consisted of all exactly geo-located Twitter posts in England from August 29, 2011 to August 30, 2015, which comprise approximately 1% of all tweets made by users in England. This number is a rough estimate based on approximately 20% of the United Kingdom population using Twitter, with 33% of active users assumed to be posting 5 tweets per day [15]. Our dataset consists of 350,000 geo-located tweets per day on average. As in Lampos et al [9], the same initial list of 36 *n*-grams (phrases with *n* words) related to ILI was created manually. Then, based on frequent cooccurrence with this list in the Twitter time series data, a set of 217 *n*-grams was extracted (*n*<5; see Multimedia Appendix 1).

The RCGP ILI rates used for model learning were only available on a weekly basis, so frequency rates of this set of *n*-grams for a period of 7 days prior to any given day were computed, and formed the explanatory variables. To estimate the impact on the pilot areas, *n*-gram frequencies of tweets geo-located in the chosen pilot and control areas during the intervention period were used.

#### Official Health Reports

Weekly ILI estimates were provided by the RCGP, a sentinel network of approximately 100 practices in England, which covers a registered population of approximately 1 million persons [12]. These ILI estimates represent the weekly incidence rate of ILI cases/consultations per 100,000 patients registered with eligible practices during that week [12]. The data used cover the period from August 29, 2011 to August 30, 2015 for England.

#### Pilot and Control Areas

A total number of 140 local authorities implemented vaccinations as part of the pilot program. To create a suitable list of pilot areas for the impact assessment, these areas were combined on a county level, where possible. This list included a large amount of *Secondary school* pilot areas (37), so only the most populated ones were considered, whilst ensuring an even geographical distribution throughout the country. The geographical distribution and the areas’ population sizes were defined using ONS boundary data and population estimates of England, respectively [13,14]. Of the 7 *Primary and Secondary school* pilot areas, 3 were eliminated due to small size or because they were enclosed within another pilot area. Pilot areas involving special schools were ignored, as these included only a small number of schools and were thus unlikely to provide any significant community-wide benefits. This preprocessing resulted in 6 *Primary school*, 4 *Primary and Secondary school*, and 7 *Secondary school* pilot areas.

**Figure 1:**
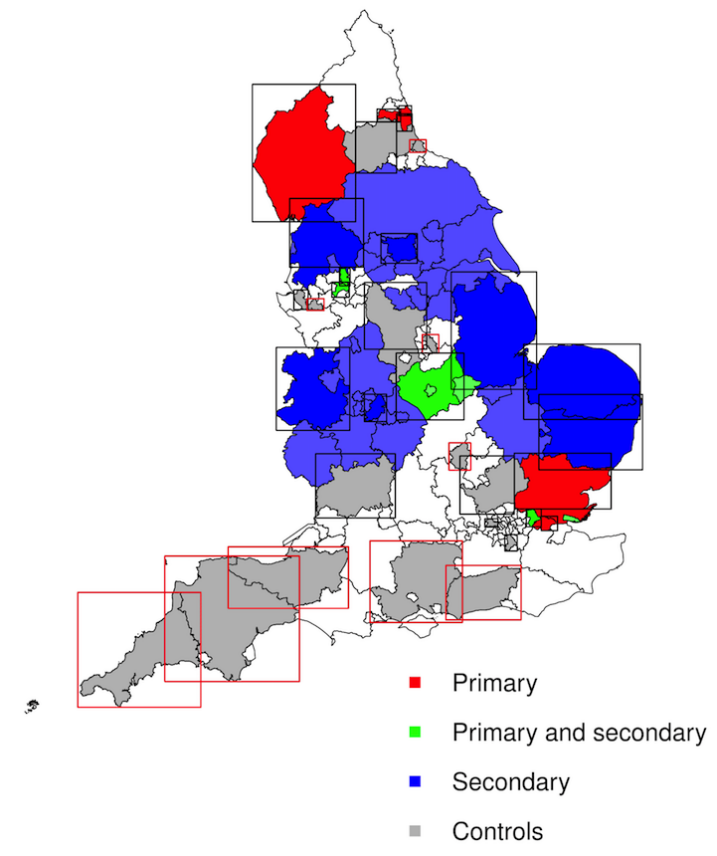
Geographical distribution of the pilot and control areas chosen for the study with their corresponding boundary boxes. Control areas with red boxes have a distance of at least 10 km to any pilot area. The “Secondary” and “Primary and Secondary” pilot areas that were excluded from the study are shown without boundary boxes and in a lighter shade of blue and green, respectively. Contains National Statistics and OS data, Crown copyright and database right.

A list of eligible control locations was chosen according to the following criteria: appropriate distance from pilot areas, a moderate population size, and a plausible geographical spread. These criteria resulted in a list of 16 control areas. Nonoverlapping boundary rectangles represented by their North-East and South-West corners were created around the chosen pilot and control areas. The geographical distribution of the pilot and control areas is shown in Figure 1. Table 1 lists the pilot areas considered for this study. For a full list of control and pilot areas, see Multimedia Appendix 2.

**Table 1:** Pilot areas considered for this study during the 2014/2015 LAIV program with their respective population size [14] and geographical boundary rectangle corner coordinates. Pilot areas that were also used or have partial overlap with the ones used in the 2013/2014 LAIV program are highlighted in italics.

Location	Pilot	Population	North-East corner^a^	South-West corner^b^
Thurrock	Primary school	163,270	51.568, 0.551	51.448, 0.334
*Gateshead*	*Primary school*	*200,505*	*54.984, -1.510*	*54.878, -1.853*
South Tyneside	Primary school	148,740	55.011, -1.352	54.928, -1.536
Sunderland	Primary school	276,889	54.944, -1.346	54.799, -1.569
*Cumbria*	*Primary school*	*497,874*	*55.189, -2.159*	*54.040, -3.641*
*Essex*	*Primary school*	*1,431,953*	*52.093, 1.297*	*51.632, -0.020*
Lancashire	Secondary school	1,184,735	54.240, -2.045	53.667, -3.085
Birmingham	Secondary school	1,101,360	52.609, -1.729	52.381, -2.034
Norfolk	Secondary school	877,710	52.993, 1.745	52.355, 0.154
Leeds	Secondary school	766,399	53.946, -1.290	53.699, -1.800
Suffolk	Secondary school	738,512	52.550, 1.769	51.932, 0.340
Lincolnshire	Secondary school	731,516	53.616, 0.358	52.640, -0.821
Shropshire	Secondary school	310,121	52.998, -2.233	52.306, -3.236
*Bury*	*Primary and Secondary school*	*187,474*	*53.667, -2.234*	*53.512, -2.383*
Salford	Primary and Secondary school	242,040	53.542, -2.245	53.416, -2.490
*Havering*	*Primary and Secondary school* ^c^	*245,974*	*51.632, 0.334*	*51.484, 0.138*
*Leicestershire*	*Primary and Secondary school*	*667,905*	*52.948, -0.664*	*52.392, -1.598*

^a^Longitude and latitude of the North-East edge of the bounding box

^b^Longitude and latitude of the South-West edge of the bounding box

^c^The secondary school program in Havering included the year 7 cohorts only (11-12 years)

### Statistical Framework

The following sections provide a brief outline of the statistical framework that was implemented. Apart from a slightly improved supervised learning approach, this framework is based on the work by Lampos et al [9], in which it is described and validated in more detail. The method consists of first learning a nonlinear regression model to estimate ILI rates from *n*-grams based on user-generated content (tweets in this case). Thereafter, by making use of inferred ILI rates in matched pilot and control regions, a linear modeling approach was applied to assess the potential impact of the intervention in the pilot areas.

#### Estimating Disease Rates Using a Gaussian Process

The majority of techniques used to acquire infectious disease estimates from user-generated data involve the use of linear regression models [16-18]. Lampos et al showed that nonlinear methods can improve model performance, especially when working with a smaller feature space consisting of varying *n*-gram sizes [8]. The authors proposed the use of Gaussian Processes (GPs) to model ILI rates and successfully applied these to Twitter, Google, and Bing data [8,9]. See below for details of the GP model used in this study.

Let **X**∈ℝ^N×^^M^ be the observation matrix with *N* weeks and *M* frequency rates of *n*-gram features. Then given inputs **x,x'**∈ℝ^M^ (representing rows of **X**), a GP can be defined as a statistical distribution for which any finite linear combination of samples is normally distributed and is written as:

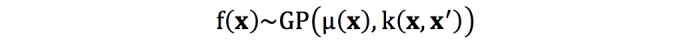



Here μ(**x**) and k(**x,x'**) represent the mean and covariance function (or kernel), respectively [19]. By assuming that μ(**x**)=0∀ *i*=1,…, *N*, the distribution is entirely determined by its covariance function. As our core kernel, the sum of two differently parameterized Matérn functions (k_M_) [20], with degrees of freedom *v*=3/2 was found to be the most suitable for estimating ILI rates from Twitter data:

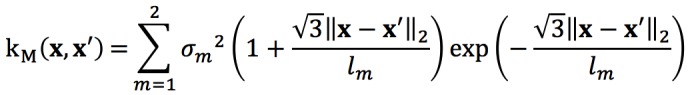



where *σ*_m_ represents the overall level of variance and *l*_m_ a characteristic length scale. Assuming that different *n*-gram sizes may vary in their usage and are likely to have a more concise semantic interpretation with an increasing *n*, we model them with different kernels. The fact that the sum of covariance functions forms a valid covariance function in itself allows for this and we have:

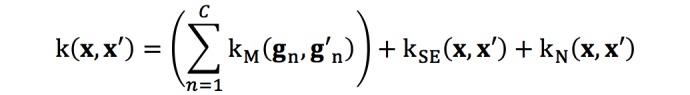



where ***g***_n_ represents the features that belong to each *n*-gram category and *C*=3 is the number of *n*-gram categories (3-grams and 4-grams are merged in this particular model). To model noise, we use the sum of a squared exponential:

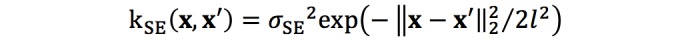



and a noise function:

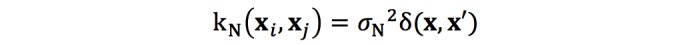



(δ is a Kronecker delta function), as defined in [19].

GP regression involves minimizing the negative log-marginal likelihood function:

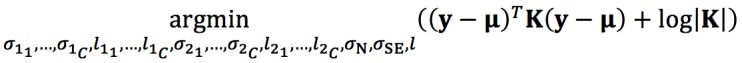



where **y** denotes the ILI rates time-series, (**K**)*_ij_*=k(**x**_i_,**x**_j_) and **μ**=(μ(**x**_1_),…,μ(**x***_N_*)). Once the model is learnt, newly observed feature frequency rates x_*_ result in new ILI rate estimates y_*_ by computing E[y_*_|**y**,Ω,x_*_], the mean of the posterior predictive distribution. The performance of the model was measured using a 10-fold cross validation (random temporal splits) on the training set, using the average Pearson correlation (*r*) and the mean absolute error (MAE).

#### Estimating the Impact of the LAIV Program

Once the GP model was trained, the impact of the LAIV campaign in pilot areas could be estimated using the methodology outlined in Lampos et al, Section 3.3 [9], which we briefly describe here as well.

Given a set of pilot and control areas, *n*-gram frequencies of Twitter posts geo-located in those areas are extracted for a period before and during the intervention. ILI rate estimates can then be computed for all areas and supersets of areas using a pretrained GP model and we denote these with **q**_v_ and **q**_c_ for pilot and control areas, respectively. By looking at these ILI estimates for a number of weeks, τ={ *t*_1_,…, *t_N_* }, prior to the intervention, control and pilot locations with similar influenza activity can be matched based on a strong Pearson correlation, 
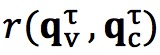
. Assuming a linear relationship in ILI rates between locations with similar influenza activity, a linear regression model can be learnt using 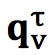
and 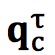
(ie, the ILI estimates prior to the intervention in the various matched area pairs):

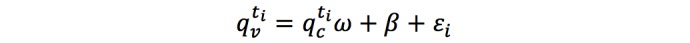



where *ω,β,ε_i_* denote the regression’s weight and intercept, and independent, zero-centered noise, respectively. Using **q**_c_, the ILI estimates in the control areas during the intervention, this linear model can then predict the hypothetical ILI rates in pilot locations during the intervention had the intervention not taken place:

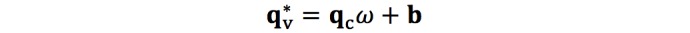



where **b**∈ℝ^N^ with (**b**)_k_= *β∀k*=1,…, *N*.

Comparing these hypothetical ILI rates to the ILI rates estimated by the GP model during the intervention allows the impact of the campaign to be estimated. The following measures were applied:

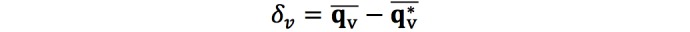

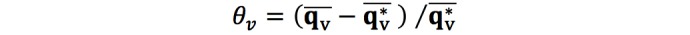





where 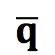
denotes the mean value of **q**. Thus, *δ_v_* and *θ_v_* measure the absolute and relative mean impact of the intervention, respectively. Confidence intervals for these measures are produced using bootstrap sampling [21]. This calculation involves sampling with replacement the residuals *ε_i_* of the linear regression, adding them to the fitted values, and then running the linear model for these, which produces estimates for *β* and *ω*. These values are then applied to a sampled (with replacement) set of **q**_v_ and **q**_c_. Repeating this procedure 100,000 times creates sets of estimates for *δ_v_* and *θ_v_* from which we can derive confidence intervals using the 0.025 and 0.975 quantiles, provided that their distributions are unimodal and symmetric. Results are considered statistically significant if absolute values are higher than two standard deviations of the bootstrap estimates [9,22].

## Results

**Figure 2:**
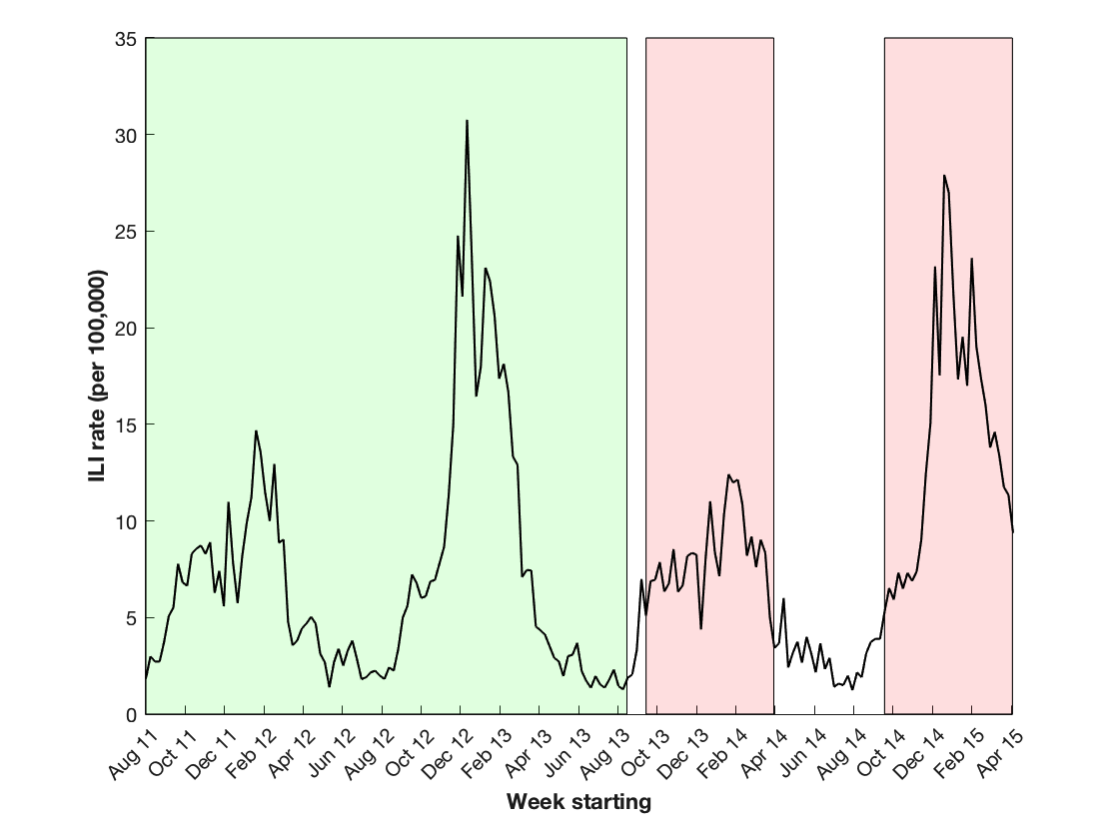
Weekly influenza-like illness (ILI) rate (per 100,000) provided by the Royal College of General Practitioners (RCGP) in England with the pre-intervention correlation period highlighted in green and the two impact assessment periods (2013/14 and 2014/15 influenza seasons) highlighted in red.

We present an assessment of the impact of the childhood LAIV campaign during the 2013/2014 and 2014/2015 influenza seasons based on the previously described methodology. The GP model was trained on RCGP ILI rates in England and Figure 2 shows the RCGP ILI rates used, with the preintervention correlation period and the two impact assessment periods highlighted.

### Performance of the Supervised Model for Estimating ILI Rates

A GP regression model was trained using weekly Twitter data geo-located in England from August 29, 2011 to August 30, 2015 and the corresponding RCGP ILI rates. Based on a 10-fold cross validation, an average Pearson correlation *r*=0.84 with a standard deviation of 0.08 and average MAE of 2.42 (weekly ILI rate per 100,000 people) with a standard deviation of 0.52 were measured. This approach is in line with the performance of the GP model used in the previous impact assessment [9].

### Impact Estimates of the LAIV Program

Using the GP model trained on a national level (England), ILI rates for the chosen pilot locations were estimated. This was done for individual pilot locations, the set of all pilot locations, and sets of pilot locations in which the same cohorts were vaccinated (ie, *Primary school*, *Secondary school*). An exhaustive search of all possible combinations of control areas was performed. These combinations of control locations were matched to the sets of pilot locations during a period prior to the start of the LAIV campaign (August 29, 2011 to September 1, 2013) based on similar influenza activity, as measured by Pearson correlation. The 2013/2014 influenza season is not included in this correlation phase, as this involved the vaccination of 2 and 3-year-olds nationally and a number of primary school age pilot areas, which could change the linear relationship between certain control and pilot locations. For each pilot area and set of pilot areas, the most highly correlated combination of control areas was used to then estimate the impact of the LAIV campaign for the 2014/2015 influenza season. There is some overlap with the pilot areas of the previous influenza season, so the same analysis was redone for the 2013/2014 season (in this case with a different set of control areas) so results could be compared to previous studies [9,11].

Table 2 and Table 3 show the results for individual pilot locations, and sets of them for the 2014/2015 and 2013/2014 influenza season, respectively. For each area, the tables include the Pearson correlation *r*, the mean and 95% confidence intervals of 100,000 bootstrap estimates of the absolute and relative mean impact δ_v_ and θ_v_ during the intervention period, the number of control areas chosen *n* (*c*), and the size of the population targeted in the pilot *Pop* (*v*) and matched collection of control *Pop* (*c*) areas. The distribution of the bootstrap estimates was assessed graphically and seemed unimodal. Thus, statistically significant results are based on absolute values being higher than two standard deviations of the bootstrap estimates and are highlighted in italics. In addition, a significant preintervention correlation was necessary for reliable impact estimates, which we defined as being a Pearson correlation >0.60, as was done in the previous study [9].

**Table 2:** Estimates of the impacts of LAIV pilot program during the 2014/2015 influenza season in individual pilot locations and supersets of them. For each area considered, the precampaign Pearson correlation *r* with chosen control areas, the mean and 95% confidence intervals of the absolute and relative mean impact δ_v_ and θ_v_ during the intervention period, the number of control areas chosen *n(c)*, and the size of the population targeted in the chosen vaccination *Pop(v)* and control *Pop(c)* areas are presented. Statistically significant results are highlighted in italics.

Pilot area	*r* ^a^	δ_v_^b^	θ_v_^c^	*n* (*c*)^d^	*Pop* (*v*)^e^	*Pop* (c)^f^
All vaccinated	0.89	-0.50 (-2.77 to 1.99)	-4.51 (-25.72 to 22.61)	10	9,772,977	5,066,069
*All “Primary school”*	*0.71*	*-1.15 (-2.19 to -0.15)*	*-16.97 (-30.09 to -2.42)*	*8*	*2,719,231*	*2,371,367*
All “Primary and Secondary school”	0.84	-0.06 (-1.50 to 1.43)	-0.30 (-16.71 to 19.36)	6	1,097,419	2,174,854
All “Primary school” and “Primary and Secondary school”	0.85	-1.35 (-3.37 to 0.66)	-13.01 (-30.54 to 7.31)	9	4,062,624	3,601,377
All “Secondary school”	0.83	0.06 (-1.58 to 1.90)	1.41 (-19.40 to 28.40)	7	5,710,353	4,038,921
Cumbria (“Primary school”)	0.59	0.04 (-0.24 to 0.33)	1.07 (-5.75 to 8.17)	7	497,874	3,999,608
Essex (“Primary school”)	0.68	-0.32 (-1.13 to 0.51)	-5.91 (-20.56 to 10.58)	8	1,431,953	3,199,730
*Gateshead (“Primary school”)*	*0.59*	*-0.39 (-0.74 to -0.04)*	*-8.46 (-15.56 to -1.02)*	*4*	*200,505*	*1,551,060*
*South Tyneside (“Primary school”)*	*0.34*	*0.25 (0.03 to 0.52)*	*6.82 (0.81 to 14.07)*	*3*	*148,740*	*1,697,971*
Sunderland (“Primary school”)	0.54	0.12 (-0.05 to 0.32)	3.20 (-1.38 to 8.38)	3	276,889	1,119,136
Thurrock (“Primary school”)	0.32	0.04 (-0.14 to 0.23)	1.01 (-3.56 to 6.24)	3	163,270	753,563
Bury (“Primary and Secondary school”)	0.32	-0.11 (-0.37 to 0.12)	-2.60 (-8.94 to 3.13)	2	187,474	893,813
Leicestershire (“Primary and Secondary school”)	0.81	0.32 (-0.70 to 1.38)	4.97 (-10.01 to 21.22)	6	667,905	2,756,865
Salford (“Primary and Secondary school”)	0.67	0.40 (-0.20 to 1.01)	8.45 (-3.96 to 22.00)	7	242,040	4,183,184
Havering (“Primary and Secondary school”-year 7)	0.48	-0.03 (-0.35 to 0.31)	-0.55 (-8.23 to 7.79)	4	245,974	1,742,705
Birmingham (“Secondary school”)	0.79	0.53 (-0.27 to 1.34)	10.36 (-4.86 to 27.21)	10	1,101,360	5,435,742
Lancashire (“Secondary school”)	0.65	0.18 (-0.78 to 1.13)	3.45 (-13.41 to 21.40)	8	1,184,735	3,463,060
Leeds (“Secondary school”)	0.63	0.54 (-0.40 to 1.51)	10.81 (-7.41 to 30.98)	7	766,399	2,731,293
Lincolnshire (“Secondary school”)	0.66	-0.29 (-0.78 to 0.19)	-6.09 (-16.20 to 4.25)	6	731,516	1,737,168
Norfolk (“Secondary school”)	0.71	-0.12 (-0.60 to 0.35)	-2.31 (-11.55 to 7.25)	6	877,710	2,784,394
Shropshire (“Secondary school”)	0.35	0.13 (-0.13 to 0.39)	3.30 (-3.18 to 9.71)	6	310,121	2,833,659
Suffolk (“Secondary school”)	0.59	0.10 (-0.34 to 0.53)	2.24 (-7.54 to 12.35)	5	738,512	2,015,339

^a^*r*: The precampaign Pearson correlation with the chosen aggregation of control areas

^b^δ_v_: The absolute difference in the mean ILI rate during the intervention period

^c^θ_v_: The relative difference in the mean ILI rate during the intervention period

^d^*n* (*c*): The number of aggregated control areas chosen

^e^*Pop* (*v*): The size of the population targeted in the chosen vaccination areas

^f^*Pop* (*c*): The size of the population targeted in the chosen aggregation of control areas

**Table 3:** Estimates of the impacts of the LAIV pilot program during the 2013/2014 influenza season in individual pilot locations and supersets of these locations. For each area considered, the precampaign Pearson correlation *r* with chosen control areas, the mean and 95% confidence intervals of the absolute and relative mean impact δ_v_ and θ_v_ during the intervention period, the number of control areas chosen *n(c)*, and the size of the population targeted in the chosen vaccination *Pop(v)* and control *Pop(c)* areas are presented. Statistically significant results are highlighted in italics.

Pilot area	*r* ^a^	δ_v_^b^	θ_v_^c^	*n* (*c*)^d^	*Pop* (*v*)^e^	*Pop* (c)^f^
*All vaccinated (Primary school)*	*0.82*	*-1.03 (-2.00 to -0.10)*	*-13.77 (-25.01 to -1.45)*	*9*	*3,231,685*	*3,601,377*
Leicestershire (Primary school)	0.81	-0.28 (-1.02 to 0.47)	-4.44 (-15.93 to 7.95)	6	667,905	2,756,865
Essex (Primary school)	0.68	0.34 (-0.30 to 1.12)	7.45 (-6.41 to 24.32)	8	1,431,953	3,199,730
Gateshead (Primary school)	0.59	0.38 (-0.06 to 0.85)	9.11 (-1.40 to 20.76)	4	200,505	1,551,060
Cumbria (Primary school)	0.59	0.36 (-0.00 to 0.75)	9.12 (-0.07 to 19.11)	7	497,874	3,999,608
Havering (Primary school)	0.48	0.15 (-0.19 to 0.52)	3.80 (-4.99 to 13.43)	4	245,974	1,742,705
Bury (Primary school)	0.32	-0.09 (-0.34 to 0.14)	-2.40 (-8.44 to 3.64)	2	187,474	893,813

^a^*r*: The precampaign Pearson correlation with the chosen aggregation of control areas

^b^δ_v_: The absolute difference in the mean ILI rate during the intervention period

^c^θ_v_: The relative difference in the mean ILI rate during the intervention period

^d^*n* (*c*): The number of aggregated control areas chosen

^e^*Pop* (*v*): The size of the population targeted in the chosen vaccination areas

^f^*Pop* (*c*): The size of the population targeted in the chosen aggregation of control areas

For the 2014/2015 influenza season, correlations ranged from 0.32 to 0.89, and pilot areas with larger populations tend to have more control areas, larger populations of control areas, and higher Pearson correlations. The only significant impact was observed in the *Primary school* age pilot areas, for which the results suggest that during the 2014/2015 influenza season the mean ILI rate was reduced by 16.97% (95% CI 2.42-30.09). For the individual locations, Gateshead and South Tyneside did show significant results, but their precampaign correlations were 0.59 and 0.34, respectively; both were less than the predefined threshold of 0.60, which makes their impact estimates possibly less reliable.

The correlations for the 2013/2014 influenza season ranged from 0.32 to 0.82, and whilst none of the individual locations demonstrated significant results, all pilots together estimated a statistically significant impact of a 13.77% (95% CI 1.45-25.01) reduction in the mean ILI rate during that season. Note that for the 2013/2014 season, the primary school-age vaccination was the only program implemented across all pilot areas.

## Discussion

### Principal Results

By using social media content to assess the impact of the childhood influenza pilot program in England in 2013/2014 and 2014/2015, statistically significant results suggest a reduction in the mean ILI rate of approximately 17% (Table 2, row 2, column 4) across all ages in *Primary school* age pilot areas only during the 2014/2015 influenza season and 14% (Table 3, row 1, column 4) in the aggregation of *Primary school* age vaccinated areas during the 2013/2014 influenza season.

### Comparison With Prior Work

Both impact estimates are in line with results from independent studies by PHE that used traditional surveillance systems [3,11]. For the 2014/2015 season, however, the impact results are generally lower than expected with only a few statistically significant results. For example, it was expected that the *Primary and Secondary school* or the combined set of *Primary school* and *Primary and Secondary school* pilot locations would yield significant impacts, as they included a similar program to that in the *Primary school* pilot areas. Looking at the boundary boxes in more detail (Figure 1) shows that of the 4 *Primary and Secondary school* pilot areas, Leicestershire and Salford both include substantial parts of nonpilot areas, which is likely to have biased their results and underestimated effect sizes. The lack of statistically significant results across all individual locations is possibly due to the sparsity of the Twitter data available. For example, the individual *Primary school* pilot areas did not yield statistically significant impact estimates (with the exception of Gateshead and South Tyneside, which did show significant results, but their preintervention correlations were below the 0.60 threshold), whilst the aggregation of all *Primary school* areas did.

The previous study by Lampos et al implemented a similar approach using Twitter and Bing data to assess the impact of the LAIV pilots during the 2013/2014 influenza season [9]. This study estimated the impact to be approximately 33% for the aggregation of all pilot locations based on Twitter data, which is more than double what was found in this study. The discrepancy between these results is most likely due to two factors. First, the pilot areas used for the 2013/2014 season in the present study are slightly larger than those in the previous one, as some of the reused pilot areas have been expanded. This issue particularly applies to the boundary boxes for Leicestershire and Essex, as the previous study only included parts of these areas. Second, apart from one control area (Liverpool), most of the previous control areas were part of the 2014/2015 pilot program, and thus not reusable. New control areas were therefore selected, which may explain the discrepancy in impact estimates. Nevertheless, given that both studies exhibited a significant impact, the methodology produces qualitatively consistent results for the same influenza season, even when using a different set of control and pilot areas.

### Conclusions

There is a strong indication that the primary school age vaccination program has the potential to be an effective strategy in reducing influenza transmission in the general population. This notion supports the ongoing rollout of the campaign for primary school children. For a secondary school-only vaccination program offering the vaccine to just two-year cohorts (and not to all children of secondary age), there is no clear evidence of any population-wide effect. Both of these conclusions are in line with findings from previous studies and complement traditional surveillance sources in exhibiting community-wide effects of the LAIV pilot campaign [3,9,11,23].

Most current influenza surveillance schemes rely on established health systems. Although these schemes provide important information on health care-related burden of disease and potential reductions due to vaccine impact, several provide less direct insight into community-wide transmission. User-generated content from social media offers rapid access to a larger range of the population, which has the potential of including a wider community (ie, including those that do not seek medical attention) and thus offers a valuable complementary source for the surveillance and evaluation of public health programs.

### Limitations

There are several potential limitations in this study. Work is still needed to refine the methods used to deal with issues such as noise, model and data biases, and the fact that estimates from user-generated content are not directly based on actual ILI cases. More advanced natural language processing techniques may deliver more accurate results [24]. The choice of control areas requires further refinement; we are seeking an even geographical distribution as well as an adequate distance from pilot areas to avoid regional biases, and to isolate the potential impact observed in pilot areas, respectively. Furthermore, the methodology is highly dependent on the quantity and type of user-generated data that is available, as this determines the accuracy and interpretation of the ILI rate estimates. The majority of Twitter users, for example, are between the ages of 15-44 years with a higher proportion situated in urban/suburban areas [25]. This factor may skew results towards illness in certain demographic groups. The current framework conducts ILI rate modeling by training on syndromic surveillance data (from RCGP), such that biases that are found there are also passed onto the models. Furthermore, even if these biases can be avoided, there is an issue that no definite ground truth exists to allow for a proper verification.

### Future Work

Future work could aim at moving towards unsupervised models that do not depend on traditional surveillance sources for training purposes. These models could produce their own, independent ILI indicators based solely on user-generated content with the potential of being able to tap into the bottom part of the disease population pyramid [10]. Inference of the demographics of users, such as age [26], socioeconomic status [27,28], or severity of disease [29] could be another focus of forthcoming work. Pebody et al showed that for both influenza seasons the impact of the pilot program was lower as influenza end-points of infection became more severe, which is an insight that the current modeling framework is unable to pick up on [3,11]. With suitable data access in the future, this framework has the potential of assessing the impact of intervention programs whose uptake is variable. The applicability of this framework extends beyond influenza, but across a number of health interventions, thereby allowing for a timely and potentially cost-effective complementary to the collection of traditional surveillance data.

## Appendix

Multimedia Appendix 2 A table of the pilot and control areas chosen with their respective population size, distance to closest pilot areas, and geographical boundary rectangle corner coordinates.

## Abbreviations

GP: Gaussian Process
ILI: influenza-like illness
LAIV: live attenuated influenza vaccine
MAE: mean absolute error
ONS: Office for National Statistics
PHE: Public Health England
RCGP: Royal College of General Practitioners

## References

[1] (2011). Joint Committee on Vaccination and Immunisation.

[2] Baguelin M, Flasche S, Camacho A, Demiris N, Miller E, Edmunds WJ (2013). Assessing optimal target populations for influenza vaccination programmes: an evidence synthesis and modeling study. PLoS Med.

[3] Pebody RG, Green HK, Andrews N, Boddington NL, Zhao H, Yonova I, Ellis J, Steinberger S, Donati M, Elliot AJ, Hughes HE, Pathirannehelage S, Mullett D, Smith GE, de Lusignan S, Zambon M (2015). Uptake and impact of vaccinating school age children against influenza during a season with circulation of drifted influenza A and B strains, England, 2014/15. Euro Surveill.

[4] Milinovich GJ, Williams GM, Clements AC, Hu W (2014). Internet-based surveillance systems for monitoring emerging infectious diseases. Lancet Infect Dis.

[5] Ginsberg J, Mohebbi MH, Patel RS, Brammer L, Smolinski MS, Brilliant L (2009). Detecting influenza epidemics using search engine query data. Nature.

[6] Lampos V, Cristianini N (2012). Nowcasting events from the social web with statistical learning. ACM Trans Intell Syst Technol.

[7] Paul MJ, Dredze M, Broniatowski D (2014). Twitter improves influenza forecasting. PLoS Curr.

[8] Lampos V, Miller AC, Crossan S, Stefansen C (2015). Advances in nowcasting influenza-like illness rates using search query logs. Sci Rep.

[9] Lampos V, Yom-Tov E, Pebody R, Cox IJ (2015). Assessing the impact of a health intervention via user-generated Internet content. Data Min Knowl Disc.

[10] Gibbons CL, Mangen MJ, Plass D, Havelaar AH, Brooke RJ, Kramarz P, Peterson KL, Stuurman AL, Cassini A, Fèvre EM, Kretzschmar MEE, Burden of Communicable diseases in Europe (BCoDE) consortium (2014). Measuring underreporting and under-ascertainment in infectious disease datasets: a comparison of methods. BMC Public Health.

[11] Pebody R, Green H, Andrews N, Zhao H, Boddington N, Bawa Z (2014). Uptake and impact of a new live attenuated influenza vaccine programme in England: early results of a pilot in primary school-age children, 2013/14 influenza season. Eurosurveillance.

[12] Correa A, Hinton W, McGovern A, van VJ, Yonova I, Jones S, de LS (2016). Royal College of General Practitioners Research and Surveillance Centre (RCGP RSC) sentinel network: a cohort profile. BMJ Open.

[13] Office for National Statistics (2011). UK Data Service.

[14] Office for National Statistics (2015). Annual mid-year population estimates.

[15] Rose K (2016). Rosemcgrory.co.uk.

[16] Lampos V, Cristianini N (2010). Tracking the influenza pandemic by monitoring the social web. 2nd International Workshop on Cognitive Information Processing.

[17] Lampos V, De Bie T, Cristianini N (2010). Flu Detector - tracking epidemics on Twitter. LNCS.

[18] Culotta A (2012). Lightweight methods to estimate influenza rates and alcohol sales volume from Twitter messages. Lang Resources & Evaluation.

[19] Rasmussen C, Williams C (2006). Gaussian processes for machine learning.

[20] Matern B (1986). Spatial variation.

[21] Efron B, Tibshirani R (1993). An introduction to the bootstrap.

[22] Lambert D, Pregibon D (2008). Online effects of offline ads.

[23] (2014). Public Health England.

[24] Lampos V, Zou B, Cox I (2017). Enhancing feature selection using word embeddings.

[25] Duggan M, Ellison N, Lampe C, Lenhart A, Madden M (2015). Pew Research Center: Internet, Science & Tech.

[26] Rao D, Yarowsky D, Shreevats A, Gupta M (2010). Classifying latent user attributes in Twitter.

[27] Preoţiuc-Pietro D, Lampos V, Aletras N (2015). An analysis of the user occupational class through Twitter content. Proceedings of the 53rd Annual Meeting of the Association for Computational Linguistics and the 7th International Joint Conference on Natural Language Processing.

[28] Lampos V, Aletras N, Geyti J, Zou B, Cox I (2016). Inferring the socioeconomic status of social media users based on behaviour and language.

[29] Yom-Tov E, Johansson-Cox I, Lampos V, Hayward AC (2015). Estimating the secondary attack rate and serial interval of influenza-like illnesses using social media. Influenza Other Respir Viruses.

