# Supplementary material for: Estimating the Population Impact of a New Pediatric Influenza Vaccination Program in England Using Social Media Content
Source: J Med Internet Res. 2017 Dec 21;19(12):e416. doi: 10.2196/jmir.8184 (PMC6257312; doi:10.2196/jmir.8184)
Supplement: Multimedia Appendix 2 [file jmir_v19i12e416_app2.pdf]

Pilot and control areas chosen with their respective population size [14], distance to closest pilot areas, and geographical boundary rectangle corner coordinates. Pilot areas that were also used or have partial overlap with the ones used in the 2013/14 LAIV programme are highlighted in bold.

Location	Pilot	Population	Nearest Pilot within (km)	NE corner[a]	SW corner[b]
Thurrock	Primary school	163,270	NA	51.568, 0.551	51.448, 0.334
**Gateshead**	**Primary school**	**200,505**	**NA**	**54.984, -1.510**	**54.878, -1.853**
South Tyneside	Primary school	148,740	NA	55.011, -1.352	54.928, -1.536
Sunderland	Primary school	276,889	NA	54.944, -1.346	54.799, -1.569
**Cumbria**	**Primary school**	**497,874**	**NA**	**55.189, -2.159**	**54.040, -3.641**
**Essex**	**Primary school**	**1,431,953**	**NA**	**52.093, 1.297**	**51.632, -0.020**
Lancashire	Secondary school	1,184,735	NA	54.240, -2.045	53.667, -3.085
Birmingham	Secondary school	1,101,360	NA	52.609, -1.729	52.381, -2.034
Norfolk	Secondary school	877,710	NA	52.993, 1.745	52.355, 0.154
Leeds	Secondary school	766,399	NA	53.946, -1.290	53.699, -1.800
Suffolk	Secondary school	738,512	NA	52.550, 1.769	51.932, 0.340
Lincolnshire	Secondary school	731,516	NA	53.616, 0.358	52.640, -0.821
Shropshire	Secondary school	310,121	NA	52.998, -2.233	52.306, -3.236
**Bury**	**Primary and Secondary school**	**187,474**	**NA**	**53.667, -2.234**	**53.512, -2.383**
Salford	Primary and Secondary school	242,040	NA	53.542, -2.245	53.416, -2.490
**Havering**	**Primary and Secondary school**[c]	**245,974**	**NA**	**51.632, 0.334**	**51.484, 0.138**
**Leicestershire**	**Primary and Secondary school**	**667,905**	**NA**	**52.948, -0.664**	**52.392, -1.598**

County Durham	Control area	517,773	0	54.878, -1.569	54.452, -2.159
Liverpool	Control area	473,073	3	53.475, -2.818	53.312, -3.019
Ealing	Control area	342,118	0	51.560, -0.245	51.490, -0.420
Croydon	Control area	376,040	0	51.423, 0.003	51.287, -0.162
Gloucestershire	Control area	611,332	0	52.113, -1.615	51.578, -2.687
Hertfordshire	Control area	1,154,766	0	52.081, -0.020	51.600, -0.746
Derbyshire	Control area	779,804	0	53.540, -1.166	52.948, -2.034
Hartlepool	Control area	92,590	12	54.727, -1.148	54.622, -1.384
Halton	Control area	126,354	10	53.403, -2.595	53.305, -2.832
Milton Keynes	Control area	259,245	18	52.196, -0.592	51.969, -0.887
Gedling	Control area	115,638	11	53.104, -1.007	52.948, -1.231
Hampshire	Control area	1,346,136	24	51.384, -0.729	50.706, -1.957
West Sussex	Control area	828,398	17	51.167, 0.045	50.722, -0.958
Devon	Control area	765,302	91	51.247, -2.887	50.201, -4.681
Cornwall	Control area	545,335	150	50.931, -4.165	49.955, -5.747
Somerset	Control area	541,609	56	51.333, -2.244	50.821, -3.840

[a] Longitude and latitude of the North-East edge of the bounding box.

[b] Longitude and latitude of the South-West edge of the bounding box.

[c] The secondary school programme in Havering included the year 7 (11-12 years) cohorts only.
